# Clinical Characteristics and Outcomes in Hospitalized Patients with Respiratory Viral Co-Infection during the 2009 H1N1 Influenza Pandemic

**DOI:** 10.1371/journal.pone.0060845

**Published:** 2013-04-09

**Authors:** Ignacio A. Echenique, Philip A. Chan, Kimberle C. Chapin, Sarah B. Andrea, Joseph L. Fava, Leonard A. Mermel

**Affiliations:** 1 Department of Medicine, Alpert Medical School of Brown University, Providence, Rhode Island, United States of America; 2 Division of Infectious Diseases, Rhode Island Hospital, Providence, Rhode Island, United States of America; 3 Department of Pathology, Rhode Island Hospital, Providence, Rhode Island, United States of America; 4 Centers for Behavioral and Preventive Medicine, The Miriam Hospital, Alpert Medical School of Brown University, Providence, Rhode Island, United States of America; University of Liverpool, United Kingdom

## Abstract

**Background:**

The clinical consequences of co-infection with two or more respiratory viruses are poorly understood. We sought to determine if co-infection with pandemic 2009–2010 influenza A H1N1 (pH1N1) and another respiratory virus was associated with worse clinical outcomes.

**Methods:**

A retrospective cohort study was performed of all hospitalized patients with a positive respiratory viral panel (RVP) for two or more viruses within 72 hours of admission at our institution from October 2009 to December 2009. We compared patients infected with one respiratory virus to those with respiratory viral co-infection.

**Results:**

We identified 617 inpatients with a positive RVP sample with a single virus and 49 inpatients with a positive RVP sample for two viruses (i.e. co-infection). Co-infected patients were significantly younger, more often had fever/chills, tachypnea, and they more often demonstrated interstitial opacities suggestive of viral pneumonia on the presenting chest radiograph (OR 7.5, 95% CI 3.4–16.5). The likelihood of death, length of stay, and requirement for intensive care unit level of care were similar in both groups, but patients with any respiratory virus co-infection were more likely to experience complications, particularly treatment for a secondary bacterial pneumonia (OR 6.8, 95% CI 3.3–14.2). Patients co-infected with pH1N1 and another respiratory virus were more likely to present with chest radiograph changes suggestive of a viral pneumonia, compared to mono-infection with pH1N1 (OR 16.9, 95% CI 4.5–62.7). By logistic regression using mono-infection with non-PH1N1 viruses as the reference group, co-infection with pH1N1 was the strongest independent predictor of treatment for a secondary bacterial pneumonia (OR 17.8, 95% CI 6.7–47.1).

**Conclusion:**

Patients with viral co-infection, particularly with pH1N1, were more likely to have chest radiograph features compatible with a viral pneumonia and complications during their hospital course, particularly treatment for secondary bacterial pneumonia. Despite this, co-infection was not associated with ICU admission.

## Introduction

In the United States, pandemic 2009–2010 influenza A (pH1N1) was first identified in April 2009 [1]. Two waves of infection followed, accounting for an estimated 61 million cases, 274,000 hospitalizations, and 12,470 deaths [2], [3]. Compared to seasonal averages, there was an increase in hospitalizations and a decrease in mortality. Children experienced a greater burden of disease and a disproportionately increased burden of mortality [4]–[10]. However, the majority of children did not progress to severe disease [11]. In contrast, fewer adults were afflicted but proportionally more experienced severe disease [12]. The clinical characteristics of pH1N1-infected individuals are well described [13]–[22].

In pediatrics, viral co-infection is frequently encountered but the clinical consequences remain unclear. Co-infection occurs in 25–40% of children with bronchiolitis [23]–[26]. Viral co-infection also increases the likelihood of requiring pediatric intensive care unit (PICU) level of care [27]. These findings may reflect certain combinations of co-infection. For example, infection with respiratory syncytial virus (RSV) and metapneumovirus is associated with a 10-fold greater likelihood of PICU level of care [28]. Although some studies revealed similar findings with RSV and rhinovirus co-infection [29]–[31], others have not confirmed this finding [32]–[37] or have found less severe disease with viral co-infection [38], [39]. In adults the clinical significance of co-infection is poorly understood. It accounts for approximately 5% (range 2%–16%) [40]–[42] of adult viral acute respiratory infections, with varying prevalence of specific pairs of viruses [43]–[47]. Co-infection during the 2009–2010 pH1N1 season varied as well [42], [48]. One study found pH1N1 co-infection with rhinovirus correlated with a lower clinical severity, whereas pH1N1 co-infection with other viruses led to greater severity [48].

Few studies have examined the clinical characteristics of co-infected patients [27], [29], [42], [49]–[54] and their outcomes [31], [43], [48], [55]–[62]. Some have described an association between pH1N1 viral co-infection and poorer outcomes [48], [57], [58], whereas others have not demonstrated differences in outcomes [55], [56], [59]–[63]. Many of these studies are limited by small sample size. Furthermore, direct comparisons are limited by varying age groups and a wide array of acuity that ranges from outpatient to exclusively critical care settings. We previously compared patients with pH1N1to those infected with other respiratory viruses [64]. In the present study, we describe the characteristics and outcomes of co-infected patients at our institution at the height of the pH1N1 pandemic.

## Materials and Methods

### Study Design

A retrospective cohort study was performed of all individuals presenting to our hospital system between October 16, 2009 and December 1, 2009 who were hospitalized and had a positive respiratory viral panel (RVP, Luminex xTAG®; Luminex Corporation, Austin, TX) within 72 hours of hospital admission. Clinical history, laboratory data, medications, radiographic imaging, and hospital course were reviewed as previously described [64]. Patients co-infected with two or more viruses, excluded from the initial study, were the focus of this analysis. We hypothesized that infection with certain combinations of respiratory viruses, particularly those with influenza pH1N1, would have worse outcomes than mono-infected patients.

Chart review was done to assess for complications such as treatment for bacterial pneumonia, aspiration pneumonia, metabolic acidosis, acute kidney injury, febrile seizure, chronic obstructive pulmonary disease exacerbation, peritonitis, and hypotension requiring vasopressors. Treatment for bacterial pneumonia was defined as reported in the discharge diagnosis, chart review, or the explicit use of antibiotics for this purpose. Antibiotics empirically started and later discontinued did not fulfill this criterion.

### Ethics Statement

The Rhode Island Hospital institutional review board approved this study. A waiver of informed consent was obtained.

### Statistical Analysis

Initial analyses examined the frequencies and percentages of categorical variables, and the means and standard deviations of continuous variables. Age was determined to be a significant covariate for many outcome variables of interest, and all subsequent analyses included age as a covariate and only the age-adjusted results are reported. Age as a variable was highly skewed and not normally distributed. Thus, a natural log transformation was used in covariate-adjusted analyses. Several continuous outcome variables (duration of symptoms pre-admission, length of intensive care unit [ICU] stay, length of hospital stay, WBC, and percent bands) were also not normally distributed and a natural log transformation was also used to normalize these variables before analysis. Logistic regression, adjusting for age, was used to examine all categorical outcomes, with results reported based on Wald tests with associated odds ratios and their 95% confidence intervals. Analysis of covariance (ANCOVA), adjusting for age, was used to examine all continuous variables. ANCOVA results report the covariate-adjusted F-test p-values and the adjusted outcome means with their standard errors and 95% confidence intervals. All adjusted natural log transformed outcome variables were transformed back into their original metric in tabled values. Analyses were performed using IBM SPSS version 20.

## Results

A total of 1,192 inpatient RVP samples were performed from October 2009 to December 2009. Six hundred and fifteen were positive for a single respiratory virus, and 52 with two viruses. No samples showed infection with three or more viruses. Review of the 52 co-infected samples revealed two samples where detection of a second virus was initially indeterminate but later finalized as negative, and were therefore reclassified as mono-infection. Additionally, a separate co-infected patient was found to have two specimens. Therefore, 617 (51.8%) inpatients with a single agent identified in their RVP were compared to 49 (4.1%) patients with co-infection (see Table 1).

**Table 1:** Respiratory viral co-infection (N = 49).

Influenza A/pH1N1 and Rhinovirus	17
Adenovirus and Rhinovirus	10
RSV-A and Rhinovirus	5
Rhinovirus and Parainfluenza IV	4
Influenza A/pH1N1 and RSV-A	2
RSV-A and Adenovirus	2
Influenza A/pH1N1 and Adenovirus	1
Influenza A/pH1N1 and Metapneumovirus	1
Influenza A/pH1N1 and Parainfluenza II	1
Influenza A/pH1N1 and Parainfluenza IV	1
Influenza A/pH1N1 and RSV-B	1
Rhinovirus and Parainfluenza I	1
RSV-A and Coronavirus (HKUI)	1
RSV-A and Parainfluenza IV	1
RSV-B and Rhinovirus	1

By uncorrected chi-square analysis, pH1N1 was identified in 49% (24/49) of the co-infected group and 47% (290/617) of the mono-infected control group (p = 0.8). No seasonal influenza A H3 or influenza B was encountered in either group. In co-infected patients, rhinovirus was observed most frequently [78% (38/49) of co-infected and 34% (208/617) of mono-infected patients, respectively (OR 6.8, 95% CI 3.4–13.6, p<0.001)]. RSV A affected 22% (11/49) of the co-infected and 5.8% (36/617) of mono-infected patients, respectively (OR 4.7, 95% CI 2.2–10.0, p<0.001). Adenovirus was present in 27% (13/49) of the co-infected and 4.4% (27/617) of mono-infected patients, respectively (OR 7.9, 95% CI 3.8–16.6, p<0.001). Parainfluenza 4 was present in 12% (6/49) and 2.3% (14/617) of the co-infected and mono-infected patients, respectively, (OR 6.0, 95% CI 2.2–16.4, p<0.001).

**Figure 1:**
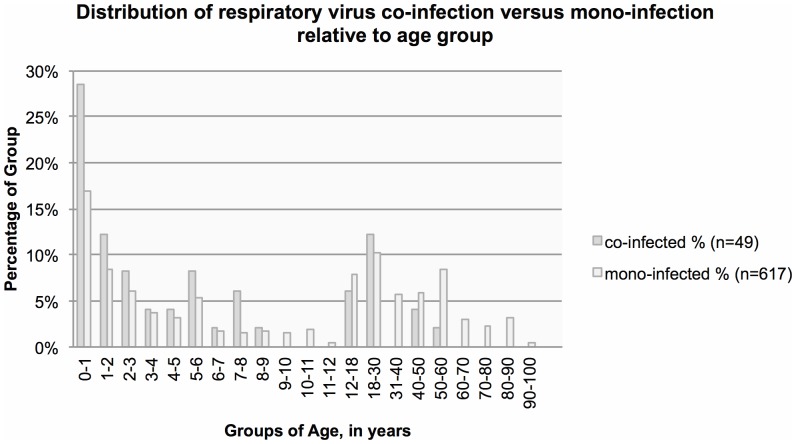
Distribution of respiratory virus co-infection versus mono-infection relative to age group.

Co-infection with any combination of respiratory viruses compared to mono-infection with any single virus was associated with younger age (mean 8.8 years of age compared to 21 years of age, respectively, p<0.001; Figure 1). To adjust for these differences, all subsequent analyses were performed with age as a covariate.

Co-infection with any combination of respiratory viruses compared to mono-infection with any single virus was associated with age less than five years (OR 3.2, 95% CI 1.5–6.8, p = 0.003; Table 1). All co-infected patients were under 60 years of age (Figure 1). Co-infected patients more frequently reported sick contacts (OR 2.0, 95% CI 1.1–3.6, p = 0.02). Co-infected patients were more frequently HIV seropositive (OR 5.5, 95% CI 1.1–27.7, p = 0.04; Table 2). Co-infected patients were more likely to present with complaints of fever/chills, and were more frequently tachypneic at presentation (36.4±1.7 breaths per minute in co-infected patients compared to 32.5±0.5 breaths per minute in mono-infected patients, p = 0.03). Both groups had similar numbers of chest radiographs (84% and 86% of co-infected and mono-infected patients, respectively). Co-infection was more often associated with interstitial opacities (OR 7.5, 95% CI 3.4–16.5, p<0.001).

**Table 2:** Age-adjusted characteristics in patients with respiratory viral co-infection compared to mono-infection.

	Co-infected (n = 49)	Mono-infected (n = 617)	Odds Ratio [95% CI]	p-value
**Age (years)**				
<5	57% (28)	38% (237)	3.2 [1.5–6.8]	.003
5 to 18	25% (12)	23% (139)	2.3 [0.95–5.6]	.07
>18^a^	18% (9)	39% (241)		
Sex (male)	67% (33)	53% (329)	1.6 [0.9–3.1]	.12
**Past Medical History**				
Sick contacts	53% (26)	34% (210)	2.0 [1.1–3.6]	.02
Visited emergency department or clinic pre-admission	57% (28)	49% (300)	1.3 [0.7–2.4]	.4
Antimicrobial agents pre-admission	18% (9)	24% (147)	0.8 [0.4–1.6]	.5
Duration of symptoms pre-admission^b^	3.0 [2.4–3.8]	2.5 [2.3–2.7]		.1
Respiratory disease	47% (23)	45% (275)	1.4 [0.7–2.5]	.3
Asthma	27% (13)	37% (230)	0.8 [0.4–1.7]	.6
Hepatic disease^c^	0.0% (0)	2.9% (18)		.6
Renal disease^c^	0.0% (0)	2.4% (15)		.2
Cancer	4.1% (2)	6.0% (37)	1.3 [0.3–5.8]	.8
Neurologic disease	8.2% (4)	11% (69)	0.8 [0.3–2.4]	.7
Cardiac disease	6.1% (3)	12.5% (77)	0.7 [0.2–2.2]	.5
Immunocompromised	8.2% (4)	6.2% (38)	2.2 [0.7–6.8]	.2
HIV	4.1% (2)	1.8% (11)	5.5 [1.1–27.7]	.04
Admission from a skilled nursing facility^c^	0.0% (0)	2.1% (13)		.7
Tobacco use (current or exposed)	8.2% (4)	16% (96)	0.7 [0.2–2.1]	.5
Pregnant^c^	0.0% (0)	1.3% (8)		.6
Patient receiving aspirin^c^	0.0% (0)	9.6% (59)		.3
**Clinical Symptoms**				
Fever/chills	92% (45)	80% (495)	3.1 [1.1–8.8]	.04
Mental status, lethargy, irritability, seizure, other neurologic disease	41% (20)	31% (189)	1.0 [0.5–1.9]	.9
Weakness	10% (5)	17% (106)	0.9 [0.3–2.6]	.9
Fatigue	8.2% (4)	15% (92)	0.6 [.2–1.7]	.3
Conjunctivitis	6.1% (3)	2.1% (13)	2.3 [0.6–8.5]	.2
Rash^c^	0.0% (0)	4.5% (28)		.02
Cough	94% (46)	88% (545)	2.4 [0.7–8.1]	.2
Productive	10% (5)	18% (113)	0.9 [0.3–2.4]	.8
Nasal symptoms	74% (36)	57% (349)	1.6 [0.8–3.2]	.2
Sore throat	8.2% (4)	24% (145)	0.4 [.1–1.2]	.1
Headache	12% (6)	20% (121)	0.9 [0.4–2.4]	.9
Myalgia	12% (6)	21% (127)	1.1 [0.4–3.0]	.9
Arthralgia	2.0% (1)	1.9% (12)	2.1 [0.3–17.3]	.5
Chest pain	12% (6)	16% (101)	1.4 [0.5–3.8]	.5
Dyspnea	74% (36)	59% (362)	1.9 [0.99–3.7]	.05
Wheezing	43% (21)	28% (173)	1.8 [0.98–3.2]	.06
Nausea	12% (6)	19% (117)	1.0 [0.4–2.6]	1.0
Vomiting	37% (18)	34% (211)	1.0 [0.5–1.8]	1.0
Abdominal pain	12% (6)	11% (66)	1.5 [0.6–3.7]	.4
Diarrhea	8.2% (4)	13% (80)	0.7 [.2–1.9]	.5
Anorexia	57% (28)	38% (232)	1.6 [0.9–3.0]	.1
**Presenting Vital Signs** d				
Initial temperature (°F)	99.9±0.3	99.7±0.1		.6
Maximum temperature (°F)	100.9±0.3	100.5±0.1		.2
Initial heart rate (/min)	131±3	130±1		.9
Maximum heart rate (/min)	136±3	136±1		1.0
Initial respiratory rate (/min)	36±2	33±1		.03
Maximum respiratory rate (/min)	39±2	36±1		.08
Admission chest X-ray Performed	84% (41)	86% (531)	1.1 [0.5–2.5]	.8
**Comparison of Chest Radiograph Results** e	**Co-infected (n = 41)**	**Mono-infected (n = 531)**	**Odds Ratio [95% CI]**	**p-value**
NAD	22% (9)	51% (273)	0.3 [0.1–0.7]	.003
IO	61% (25)	16% (86)	7.5 [3.4–16.5]	<.001
FASD	29% (12)	20% (105)	1.7 [0.8–3.4]	.1
MFASD^c^	0% (0)	9.6% (51)		.05
Edema^c^	0% (0)	3.0% (16)		.6
Effusion^c^	0% (0)	1.9% (10)		.5
Pneumomediastinum^c^	0% (0)	0.6% (3)		.6
Collapse^c^	0% (0)	0.2% (1)		.8
**Lab Results**	**Co-infected (n = 38)**	**Mono-infected (n = 435)**		
WBC^b^	9.4 [7.9–11.2]	9.2 [8.8–9.7]		.9
	**Co-infected (n = 38)**	**Mono-infected (n = 424)**		
Percent bands^b^	0.9 [0.4–1.6]	1.0 [0.8–1.2]		.7

a Reference category.

b Back transformation of the mean age-adjusted natural log values analyzed, along with back transformed 95% confidence intervals.

c Odds ratios not computed on variables with zero occurrences in a cell category.

d Adjusted means and standard errors are presented.

e NAD: No acute disease; IO: interstitial opacities; FASD: focal airspace disease; MFASD: multifocal airspace disease.

Once hospitalized, oseltamivir was used more often in co-infected than mono-infected patients [80% (39/49) and 62% (385/617), respectively, OR 3.3, 95% CI 1.6–7.0, p = 0.001]. More co-infected patients received antibacterial agents compared to mono-infected patients [76% (37/49) and 55% (337/617), respectively, OR 3.1, 95% CI 1.6–6.2, p = 0.001, Table 3].

**Table 3:** Age-adjusted treatments and outcomes in patients with respiratory viral co-infection compared to mono-infection.

	Co-infected (n = 49)	Mono-infected (n = 617)	Odds Ratio [95% CI]	p-value
**Treatment**				
Oseltamivir	80% (39)	62% (385)	3.3 [1.6–7.0]	.001
Zanamivir (inhaled)	2.0% (1)	0.2% (1)	24 [1.4–400.5]	.03
Peramivir^a^	0% (0)	0.5% (3)		.7
Ribavirin^a^	0% (0)	0.2% (1)		.9
Antibiotics	76% (37)	55% (337)	3.1 [1.6–6.2]	.001
Steroids	53% (26)	41% (252)	1.9 [1.03–3.4]	.04
Admissions to any ICU	25% (12)	17% (104)	1.6 [0.8–3.2]	.2
ICU length of stay^b^	3.5 [2.1–5.7]	2.9 [2.5–3.4]		.5
Intubation	8.2% (4)	3.7% (23)	2.8 [0.9–8.8]	.07
Positive airway pressure	2.0% (1)	3.6% (22)	1.0 [0.1–7.9]	1.0
Hi-flow nasal cannula	16% (8)	9.1% (56)	1.4 [0.6–3.3]	.4
Vasopressor use	4.1% (2)	1.8% (11)	3.2 [0.7–15.5]	.2
Nebulizers or inhalers	63% (31)	53% (324)	1.6 [0.9–2.9]	.1
**Outcome**				
Hospital length of stay^b^	3.3 [2.7–4.0]	2.8 [2.6–2.9]		.1
Complications	37% (18)	23% (142)	3.5 [1.8–7.0]	<.001
Treatment for bacterial pneumonia alone	31% (15)	9.2% (57)	6.8 [3.3–14.2]	<.001
Death	2.0% (1)	1.1% (7)	4.0 [0.4–35.2]	.2

a Odds Ratios not computed on variables with zero occurrences in a cell category.

b Back transformation into days, of the mean age-adjusted natural log values analyzed, along with the back transformed 95% confidence intervals. Analysis conducted on data available on 114 of 116 admitted to ICU.

Among co-infected patients, 15 (31%) were treated for a potential bacterial pneumonia, 4 (8.2%) had respiratory isolates sent for analysis, with confirmation of a bacterial pneumonia in one patient (2.0%). In contrast, 57 (9.2%) mono-infected patients were treated for a potential bacterial pneumonia. Respiratory isolates were obtained in 60 patients (9.7%), with identification of a causative pathogen in 17 (2.8%). An additional three patients had *Streptococcus pneumoniae* bacteremia. Co-infected patients were more likely to experience complications (OR 3.5, 95% CI 1.8–7.0, p<0.001), particularly treatment for a secondary bacterial pneumonia (OR 6.8, 95% CI 3.3–14.2, p<0.001). Most (72%) patients treated for a secondary bacteria pneumonia were infected with pH1N1.

**Figure 2:**
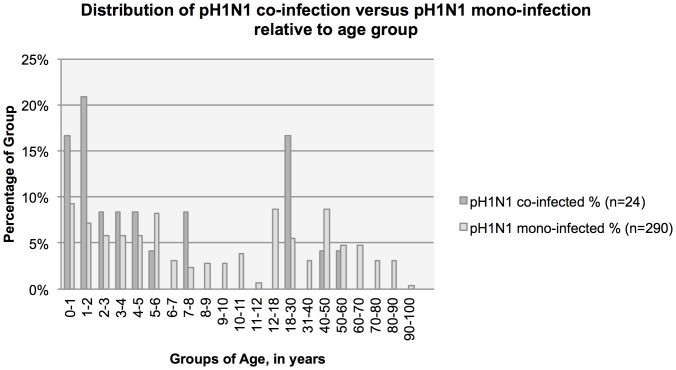
Distribution of pH1N1 co-infection versus pH1N1 mono-infection relative to age group.

Further analysis was performed of patients co-infected with pH1N1 and another respiratory virus (n = 24) compared with pH1N1 mono-infection (n = 290). Of patients co-infected with pH1N1, 71% had rhinovirus, 8.3% RSV A, 4.2% RSV B, 4.2% adenovirus, 4.2% metapneumovirus, 4.2% parainfluenza II, and 4.2% with parainfluenza IV. Co-infected pH1N1 patients, when compared to mono-infected pH1N1 patients were younger (mean age of 14 years and 23 years, respectively, p = 0.04; Figure 2). Because of the unequal distribution of age, we performed all subsequent analyses with age as a covariate. Once performed, pH1N1 co-infection, as compared to pH1N1 mono-infection, was not significantly associated with any age category.

PH1N1 co-infected patients were more likely to be immunocompromised, particularly with HIV infection. Co-infected pH1N1 patients more often complained of dyspnea and symptoms consistent with conjunctivitis. Co-infected pH1N1 patients were more likely to have interstitial opacities on their admission chest radiograph (Table 4).

**Table 4:** Age-adjusted characteristics in patients with pH1N1 influenza viral co-infection compared to pH1N1 mono-infection.

	Co-infected (n = 24)	Mono-infected (n = 290)	Odds Ratio [95% CI]	p-value
**Age (years)**				
<5	29% (7)	21% (62)	2.2 [0.8–6.7]	.1
5 to 18	42% (10)	31% (89)	2.2 [0.8–6.1]	.1
>18^a^	29% (7)	48% (139)		
Sex (male)	63% (15)	53% (153)	1.4 [0.6–3.3]	.5
**Past Medical History**				
Sick contacts	54% (13)	43% (124)	1.4 [0.6–3.2]	.5
Visited an emergency department or clinic pre-admission	54% (13)	49% (143)	1.1 [0.5–2.6]	.8
Antimicrobial agents pre-admission	17% (4)	24% (69)	0.6 [0.2–1.8]	.4
Duration of symptoms pre-admission^b^	3.2 [2.4–4.4]	2.4 [2.2–2.6]		.06
Respiratory disease	54% (13)	47% (135)	1.5 [0.6–3.5]	.4
Asthma	33% (8)	40% (115)	1.0 [0.4–2.4]	.9
Hepatic disease^c^	0% (0)	4.1% (12)		.5
Renal disease^c^	0% (0)	2.4% (7)		.4
Cancer	8.3% (2)	2.8% (8)	3.7 [0.7–19.3]	.1
Neurologic disease	4.2% (1)	13% (37)	0.3 [0.04–2.2]	.2
Cardiac disease	8.3% (2)	9.7% (28)	1.1 [0.2–5.0]	.9
Immunocompromised	17% (4)	4.8% (14)	5.5 [1.6–19.6]	.008
HIV	8.3% (2)	1.4% (4)	11.2 [1.8–70.8]	.010
Admitted from skilled nursing facility^c^	0% (0)	0.7% (2)		.8
Tobacco use	13% (3)	21% (61)	0.6 [0.2–2.2]	.5
Pregnant^c^	0% (0)	1.4% (4)		.6
Patient on aspirin^c^	0% (0)	7.6% (22)		.4
**Clinical Symptoms**				
Fever/chills^c^	100% (24)	92% (266)		.2
Mental status, lethargy, irritability, seizure, other neurologic disease	17% (4)	25% (72)	0.3 [0.1–1.03]	.06
Weakness	17% (4)	27% (78)	0.8 [0.2–2.5]	.7
Fatigue	4.2% (1)	20% (57)	0.2 [.02–1.4]	.1
Conjunctivitis	8.3% (2)	1.0% (3)	7.3 [1.1–47.2]	.04
Rash^c^	0.0% (0)	4.5% (13)		.1
Cough	96% (23)	91% (264)	2.5 [0.3–19.7]	.4
Productive	17% (4)	23% (66)	1.1 [0.3–3.6]	.9
Nasal symptoms	58% (14)	57% (164)	0.9 [0.4–2.1]	.8
Sore throat	8.3% (2)	32% (92)	0.2 [.1–1.02]	.05
Headache	21% (5)	30% (86)	0.8 [0.3–2.3]	.7
Myalgia	21% (5)	32% (92)	0.8 [0.3–2.5]	.7
Arthralgia^c^	0% (0)	3.1% (9)		.6
Chest Pain	25% (6)	23% (68)	1.6 [0.6–4.5]	.4
Dyspnea	75% (18)	53% (153)	3.0 [1.1–7.8]	.03
Wheezing	38% (9)	26% (74)	1.7 [0.7–4.0]	.2
Nausea	25% (6)	31% (91)	1.0 [0.4–2.9]	1.0
Vomiting	54% (13)	39% (113)	1.7 [0.7–3.9]	.2
Abdominal pain	17% (4)	15% (42)	1.2 [0.4–3.8]	.7
Diarrhea	13% (3)	16% (46)	0.8 [.2–2.8]	.7
Anorexia	50% (12)	38% (111)	1.2 [0.5–3.0]	.7
**Presenting Vital Signs** d				
Initial temperature (°F)	99.7±0.4	99.9±0.1		.6
Maximum temperature (°F)	101.0±0.4	100.9±0.1		.9
Initial heart rate (/min)	124±5	123±1		.9
Maximum heart rate (/min)	136±4	129±1		.2
Initial respiratory rate (/min)	31±2	28±1		.1
Maximum respiratory rate (/min)	34±2	32±1		.3
Admission chest plain film performed	83% (20)	88% (256)	0.8 [0.3–2.6]	.7
**Comparison of Chest Radiograph Results** e	**Co-infected (n = 20)**	**Mono-infected (n = 256)**	**Odds Ratio [95% CI]**	**p-value**
NAD	25% (5)	59% (152)	0.3 [0.1–0.7]	.01
IO	55% (11)	11% (27)	16.9 [4.5–62.7]	<.001
FASD	30% (6)	20% (51)	1.6 [0.6–4.5]	.340
MFASD^c^	0% (0)	7.4% (19)		.2
Edema^c^	0% (0)	2.3% (6)		.7
Effusion^c^	0% (0)	1.6% (4)		.6
Pneumomediastinum^c^	0% (0)	0.8% (2)		.6
Collapse^c^	0% (0)	0.4% (1)		.7
**Lab Results**	**Co-infected (n = 20)**	**Mono-infected (n = 245)**		
WBC^b^	7.6 [6.2–9.3]	7.7 [7.3–8.2]		.9
	**Co-infected (n = 20)**	**Mono-infected (n = 240)**		
Percent bands^b^	1.0 [0.3–2.1]	1.0 [0.7–1.2]		1.0

a Reference category.

b Back transformation of the mean age-adjusted natural log values analyzed, along with back transformed 95% confidence intervals.

c Odds ratios not computed on variables with zero occurrences in a cell category.

d Adjusted means and standard errors are presented.

e NAD: No acute disease; IO: interstitial opacities; FASD: focal airspace disease; MFASD: multifocal airspace disease.

Patients co-infected with pH1N1 were more likely to experience complications and to receive treatment for a secondary bacterial pneumonia (OR 6.3, 95% CI 2.5–15.8, p<0.001; Table 5).

**Table 5:** Age-adjusted treatments and outcomes in patients with pH1N1 influenza viral co-infection compared to pH1N1 mono-infection.

	Co-infected (n = 24)	Mono-infected (n = 290)	Odds Ratio [95% CI]	p-value
**Treatment**				
Oseltamivir	92% (22)	79% (229)	3.7 [0.8–16.5]	.09
Zanamivir (inhaled)	4.2% (1)	0.3% (1)	18.2 [1.1–310.3]	.05
Peramivir^a^	0% (0)	1.0% (3)		.7
Ribavirin^a^	0% (0)	0% (0)		–
Antibiotics	79% (19)	55% (160)	3.2 [1.2–8.9]	.03
Steroids	63% (15)	34% (99)	4.1 [1.7–10.2]	.002
Admissions to any ICU	25% (6)	16% (47)	1.9 [0.7–5.0]	.2
ICU length of stay^b^	3.0 [1.6–5.8]	3.4 [2.7–4.3]		.7
Intubation	8.3% (2)	3.4% (10)	3.3 [0.7–16.9]	.1
Positive airway pressure	4.2% (1)	5.2% (15)	1.0 [0.1–8.4]	1.0
Hi-flow nasal cannula	13% (3)	6.9% (20)	1.7 [0.5–6.3]	.4
Vasopressor use	8.3% (2)	2.8% (8)	4.4 [0.8–23.0]	.08
Nebulizer or inhaler use	63% (15)	47% (135)	2.2 [0.9–5.2]	.09
**Outcome**				
Hospital length of stay^b^	3.0 [2.2–4.0]	2.6 [2.4–2.8]		.4
Complications	54% (13)	38% (110)	2.7 [1.1–6.7]	.03
Treatment for bacterial pneumonia alone	46% (11)	14% (41)	6.3 [2.5–15.8]	<.001
Death	4.2% (1)	2.1% (6)	3.3 [0.4–30.7]	.3

a Odds Ratios not computed on variables with zero occurrences in a cell category.

b Back transformation into days, of the mean age-adjusted natural log values analyzed, along with the back transformed 95% confidence intervals. Analysis conducted on data available on 114 of 116 admitted to ICU.

Using logistic regression with the reference group composed of mono-infected patients other than pH1N1, all co-infected groups had an increased likelihood of treatment for a secondary bacterial pneumonia, particularly co-infection with pH1N1 (OR 17.8, 95% CI 6.7–47.1). Increasing age was also associated with such treatment (OR 1.5, 95% CI 1.2–1.88, p<0.001; Table 6).

**Table 6:** Independent predictors of treatment for a secondary bacterial pneumonia comparing patients with non-pH1N1 mono-infection to other patient groups.

Group	Odds Ratio [95% CI]	p-value
pH1N1 alone	2.7 [1.5–4.9]	0.002
Co-infected, not pH1N1	6.0 [1.7–20.9]	0.005
Co-infected, pH1N1	17.8 [6.7–47.1]	<0.001
Gender	1.1 [0.7–1.9]	0.7
Age^a^	1.5 [1.2–1.9]	<0.001

a Age as Nat log (age +1) to adjust for significant variance at the group level and between groups.

## Discussion

We found 7.4% of hospitalized patients with a positive respiratory viral panel had co-infection, similar to other studies [41], [42], [48], [55], [65]. While there were distinct differences in presentation, we did not find a specific prodrome to distinguish respiratory virus co-infection from mono-infection. PH1N1 co-infected patients were more likely to present with interstitial opacities consistent with a viral pneumonia and they were more likely to received treatment for a presumed secondary bacterial pneumonia. However, there were no differences in admission to any ICU, ICU length of stay, or duration of hospitalization. These findings appear incongruent, as other authors have described an association between pH1N1 mono-infection and secondary bacterial pneumonia, which in turn is associated with increased morbidity and mortality [14], [19], [42], [47], [49], [66]–[73]. We used the treatment for a bacterial pneumonia as a surrogate marker for this complication. Only a third of patients treated for a bacterial pneumonia had respiratory specimens submitted. Thus, our ability to microbiologically confirm this diagnosis was limited. Additionally, the misinterpretation of interstitial opacities on admission chest radiographs as representative of bacterial rather than viral pneumonia likely contributed to provider overtreatment.

Overall, we observed a higher frequency of interstitial opacities consistent with viral pneumonia in both co-infection in general, but also with pH1N1 co-infection specifically. There is increasing recognition of the various forms of viral pneumonia associated with pH1N1 [74]–[83] To our knowledge, only one other study has described the association between respiratory virus co-infection and an increased likelihood of a viral pneumonia [60]. The dearth of deep respiratory specimens limits the interpretation of our findings, but the radiographic and clinical characteristics of our patients support the association between respiratory virus co-infection and viral pneumonia.

Co-infection occurred more frequently in younger patients and the likelihood of receiving treatment for a secondary bacterial pneumonia increased with increasing age. Of note, we did not identify any patients with respiratory viral co-infection greater than sixty years of age. This may be secondary to the younger age distribution of our cohort or may be due to other immunologic or host parameters in the aging population in general or particular to pH1N1 [84], [85]. Younger patients may have an absence of protective antibodies or other forms of immunity from limited past exposure to viral pathogens, making co-infection potentially more likely.

While studies during previous seasons have reported a similar likelihood of co-infection as we observed, many studies were limited to the critical care or outpatient setting which may introduce selection bias by virtue of patient acuity [22], [26], [33]–[37], [40], [43], [46]. While hospitalized patients with respiratory virus co-infection did not experience poorer outcomes in our study, our findings do not address whether it was a risk factor for hospitalization itself. To this end, a large multi-center study across various levels of care is necessary.

In influenza mono-infection, the host response is simultaneously pro- and anti-inflammatory [86]. Exceeding these bounds, pH1N1, as compared to seasonal influenza, demonstrates an accentuated pro-inflammatory response, but also a suppressed adaptive immune cytokine response [87]–[91]. The pathogenesis of dual respiratory viral infections is unclear. Esper et al found co-infection with pH1N1 and rhinovirus correlated with lower clinical severity, whereas other pH1N1 virus pairs had greater severity, independent of pH1N1 titers [48]. Elsewhere, co-infection with RSV and another virus was associated with a decreased IFN-gamma response and ultimately increased severity [31]. Further research into the host cytokine and cellular responses of co-infected patients are needed, as are studies with a more robust microbiologic assessment to distinguish viral from bacterial pneumonia.

## Conclusion

Respiratory virus co-infection may be associated with differences in disease manifestation and complications, particularly chest radiographic changes suggestive of viral pneumonia and treatment for a presumed secondary bacterial pneumonia. Even when adjusted for pH1N1, which has a known association with bacterial pneumonia, co-infection in all forms was associated with treatment for a bacterial pneumonia. Co-infection with pH1N1 in particular carries the greatest risk for this complication. However, our findings suggest that respiratory virus co-infection is not associated with worse outcomes despite these complications.
